# Quantifying the impact of coefficient of thermal expansion of overlay concrete on unbonded concrete overlay performance

**DOI:** 10.1016/j.heliyon.2018.e00855

**Published:** 2018-10-18

**Authors:** Gauhar Sabih, Tahmidur Rahman, Rafiqul A. Tarefder

**Affiliations:** a Department of Civil Engineering, University of New Mexico, Albuquerque, NM 87131, USA; b Fugro USA Land Inc, 8613 Cross Park Dr. Austin, Texas 78754, USA

**Keywords:** Civil engineering

## Abstract

With the advancement in pavement design and performance analysis procedures, the coefficient of thermal expansion (CTE) of concrete has emerged as a significant design input with a direct impact on concrete pavement performance parameters including transverse cracking, joint faulting, and pavement roughness. CTE is the measure of change in concrete volume with temperature change and the resulting curling of concrete pavement slab is directly proportional to CTE. Un-Bonded Concrete Overlay (UBCO) is a cost-effective and sustainable rehabilitation technique on Jointed Plain Concrete Pavements (JPCP) to improve the performance of deteriorated concrete pavements. This study examines the effects of variability of CTE on the performance of unbonded JPCP overlays for two different climatic regions. Simulations were conducted using AASHTO pavement ME design software with varying CTE values in the range of 6.8–10.8 micro-strain/°C and keeping all other design variables as constant. The performance predictions were evaluated for different values of CTE and the results indicated that with an increase in CTE value, the performance of UBCO is adversely affected by the increase in pavement distresses. Amongst all the performance parameters, transverse cracking is the most significantly affected parameter with the change in CTE. The impact of geometric properties of overlay pavement including transverse joint spacing and slab thickness on the pavement performance was also analyzed which indicated that these have a direct impact on the performance parameters. The overlay performance can be improved by increased overlay slab thickness or reduced joint spacing and with these modifications, the adverse effects of higher CTE can be compensated. Field performance data of UBCO extracted from the LTPP database showed that the pavement ME design software can accurately predict the performance of UBCO pavement systems.

## 1 Introduction

The massive increase in highway traffic volume across the globe has led to the early deterioration of highway pavements and a greater reliance on the rehabilitation techniques of existing distressed/damaged pavements. Jointed plain concrete pavement (JPCP) is a widely used rigid pavement type in the United States and unbonded concrete overlay (UBCO) is a cost-effective rehabilitation technique to improve the performance of the existing deteriorated JPCP. UBCO is a sustainable solution for improved management of pavement assets and the primary purpose of an unbonded overlay is to restore the structural capacity of an existing deteriorated/damaged pavement. UBCO involves placement of an inter-layer of hot mix asphalt (HMA), which acts as a separation layer between the existing concrete pavement and the unbonded overlay. It is also called the de-bonding layer and is usually a thin HMA layer of 25.4–50.8 mm (1–2 inch) thickness. Factors that affect the performance of UBCO include traffic volume, climate, concrete material properties, existing pavement condition, and design life. The latest pavement performance prediction tools such as the pavement mechanistic-empirical (ME) design has provided the opportunity to analyze the performance of concrete pavements rehab designs. Wholesome literature is available on the effects of various concrete properties on the performance of concrete pavements but there has not been much research on the performance analysis of UBCOs. In a recent study of the sensitivity of various material properties on rigid pavement performance, Schwartz et al. [1] identified, the coefficient of thermal expansion (CTE) as a significant factor having a significant impact on the concrete pavement's performance.

McCarthy et al. [2] conducted their research on the accuracy of CTE inputs and found that a precision of ±0.9 micro-strain/°C (±0.5 micro-strain/°F) has a significant impact on the service life of JPCP in terms of the number of years before the requirement of rehabilitation. Rao et al. [3] conducted a study on curling in JPCP based on temperature conditions with field data collected from fully instrumented sections in Arizona and Minnesota, including the temperature data through slab thickness at different times of the day. Based on the data analysis, they concluded that actual temperature gradients have to be considered in pavement analysis. Olga Selezneva et al. [4] identified the material characteristics of concrete including strength and CTE as the critical design factors that affect the structural performance of concrete pavements. Vandenbossche, Mu, and Burnham [5] evaluated the effects of concrete material properties and pavement structural parameters on slab cracking predictions and suggested that small changes in input values of CTE can lead to significant changes in predicted performance. Sabih and Tarefder [6] investigated the impact of mechanical and thermal properties of concrete on mechanistic-empirical performance predictions of JPCP and found that along with other concrete properties, CTE significantly affects the performance of rigid pavements over the pavement service life.

The CTE of Paving mixes is tested according to AASHTO T-336 test protocol [7] and ranges from about 7.2 to 14.4 micro-strain/°C (4–8 micro-strain/°F) for the concrete cast with different coarse aggregates. The magnitudes of temperature related pavement deformations commonly known as curling are directly related to the CTE of paving concrete. Using an erroneous CTE value may lead to inaccuracy in rigid pavement design [8].

The structural parameters of JPCP which is a single layer concrete pavement and UBCO are different as the JPCP involves placement of concrete slab over the unbound layers whereas UBCO comprises the overlay concrete slab over the cracked concrete pavement with a flexible HMA layer between the two. The behavior of the two pavements to the temperature and wheel loads should be entirely different based on their structure especially with the insertion of the flexible interlayer in UBCO. The available literature is focused on the effects of CTE on single layer concrete pavements and there was a need to evaluate the impact of CTE on the performance of UBCO. This study focuses on the effects of CTE variation on the performance of unbonded JPCP overlay of existing JPCP. In this study, simulations were conducted in Pavement ME design version 2.3 for two climatic regions with different CTE values of overlay concrete and keeping all other material, traffic and other design parameters as constant to analyze the impact of CTE variability on the performance over the design life of unbonded JPCP overlays. The two climatic regions were considered to observe the effects of CTE along with different climatic conditions. Modifications in pavement structural/geometric properties were considered to minimize the effects of CTE on overlay performance. The objectives of this study are as under:

• Quantifying the effects of CTE inputs on the performance of unbonded JPCP overlays including joint faulting, transverse cracking, and pavement roughness for two different climatic regions.
• Mitigating the effects of higher CTE on overlay performance by modifying the geometric properties of the overlay pavement.

## 2 Background

The pavement ME design procedure was initially developed under the National Cooperative Highway Research Program (NCHRP) project 1-37A and further revised under continuing research projects. AASHTO eventually adopted the procedure as the standard for pavement design in 2008 [9]. Rigid pavement analysis and design including unbonded overlays are conducted using the software program known as Pavement ME design [10]. In the design process, the pavement responses such as stresses, strains, and deflections under axle loads and climatic conditions are calculated. The calculated damage of the pavement is then empirically related to pavement distresses based on the field data of the performance of in-service pavements. It helps simplify the pavement design process and results in improved rigid pavement design [11].

### 2.1 Performance indicators for unbonded JPCP overlays

The performance indicators for unbonded JPCP overlays are joint faulting, transverse cracking and pavement roughness. The threshold and reliability limits for each of the performance indicator is required to be set by the pavement design engineer. The ME design default performance criteria were selected for the analysis of the results of this study which is tabulated in Table 1 [12].

**Table 1:** Performance threshold criteria.

Performance criteria	Threshold limit
Initial IRI (cm/km)	99
Terminal IRI (cm/km)	271
Transverse cracking (% slabs)	15
Mean joint faulting (mm)	3

#### 2.1.1 Transverse cracking

This parameter is a combination of top-down and the bottom-up transverse cracks. The overlay pavement slab experiences two types of curling depending on the temperature gradient through the slab thickness commonly known as upward curling and downward curling. Upward curling occurs when there is a positive temperature gradient in the slab (usually during daytime) whereas the downward curling occurs when there is a negative temperature gradient in the overlying slab. Bottom-up cracking occurs due to the downward curling and top-down cracking is due to the upward curling. Major factors that affect transverse cracking are CTE of overlay concrete, slab thickness, joint spacing, and concrete strength. In Pavement ME Design, the transverse slab cracking predictions are calculated from a set of equations as follows [9]:

(1) $$\log\left(N_{allowable}\right)=C_{1}\left(\frac{MOR}{\sigma }\right)C_{2}$$

(2) $$Crack=\frac{100}{1+C_{4}*{\left(\frac{N_{applied}}{N_{allowable}}\right)}^{C_{5}}}$$

where *MOR* = Modulus of rupture of the concrete; *σ* = Critical stress in the slab; *N*_*applied*_ = Applied number of load applications; N_allowable_ = Allowable number of load applications; *C*_*1*_*, C*_*2*_*, C*_*4*_*, C*_*5*_ = Calibration coefficients.

Total transverse cracking predictions are calculated as follows [9].

(3) $$T_{crack}=\left(Crack_{bottom-up}+Crack_{top-down}-Crack_{bottom-up}*Crack_{top-down}\right)*100$$

where *T*_*crack*_ = Total transverse cracking (percent); *Crack*_*Bottom-up*_ = Predicted amount of bottom-up transverse cracking (fraction); *Crack*_*Top-down*_ = Predicted amount of top-down transverse cracking (fraction).

#### 2.1.2 Joint faulting

Transverse joint faulting measures the differential elevation across the transverse joint. Pavement ME design predicts the mean faulting of all the transverse joints in a pavement section and the unit of joint faulting is mm or inch. Joint faulting is directly proportional to upward curling of the overlying slab that is dependent on the CTE of overlay concrete. The upward curling results in the creation of void spaces beneath the slab edges and with the repeated heavy axle load the erosion of the sublayer starts. This erosion causes the faulting of the slab edges. Transverse joint faulting is calculated from the following set of equations [9]:

(4) $$Fault_{m}=\sum_{i=1}^{m}\Delta Fault_{i}$$

(5) $${\Delta Fault_{i}=C_{34}*\left(FaultMax_{i-1}-Fault_{i-1}\right)}^{2}*DE_{i}$$

(6) $$FaultMax_{i}=FaultMax_{i-1}+\left(C_{7}/10^{6}\right)\sum_{j=1}^{m}{DE_{j}*\log\left(1+C_{5}*5^{Erod}\right)}^{C_{6}}$$

(7) $$FaultMax_{0}=C_{12}*\delta_{curling}*[\log\left(1+C_{5}*5^{Erod}\right)*\log{\left(\frac{P_{200}*Wetdays}{P_{s}}\right)]}^{C_{6}}$$

where Fault_m_ = Mean joint faulting at the end of month m; ΔFAULT_i_ = Incremental change (monthly) in mean joint faulting during month I; FAULTMAX_i_ = Maximum mean transverse joint faulting for month I; FAULTMAX_0_ = Initial maximum mean transverse joint faulting; EROD = Base/subbase erodibility factor; DE_i_ = Differential density of energy of subgrade deformation accumulated for month I; δ_curling_ = Maximum mean monthly slab corner upward deflection in PCC due to temperature curling and moisture warping; Ps = Overburden on subgrade; P_200_ = Percent subgrade material passing #200 sieve; Wet Days = Average annual number of wet days; C1, 2, 3, 4, 5, 6, 7, 12, 34 = Calibration coefficients.

#### 2.1.3 Pavement roughness

Pavement roughness can be defined as an expression of irregularities in the overlay surface that adversely affect the ride quality. Roughness affects the ride quality, vehicle delay costs, fuel consumption and maintenance costs. Roughness is quantified using international roughness index (IRI). It is the characteristic of the longitudinal profile of a traveled wheel-track and constitutes a standardized roughness measurement [12]. The units of pavement roughness are cm/km or in/mile. Pavement roughness is a function of transverse cracking and joint faulting along with other factors thus it is evident from the previous discussion that roughness is also a function of CTE. IRI prediction equation is as follows [9]:

(8) $$IRI=IRI_{ini}+C_{1}*Crack+C_{2}*Spall+C_{3}*\frac{TFault*5280}{JSP}+C_{4}*SF$$

Where *IRI* = Predicted IRI; *IRI*_*ini*_ = Initial smoothness measured as IRI; *CRK* = Percent slabs with transverse cracks (all severities); *SPALL* = Percentage of joints with spalling (medium and high severities); *TFAULT* = Total joint faulting cumulated; *SF =* Site factor; *C*_*1, 2, 3, 4*_ = Calibration coefficients; *JSP* = Joint spacing.

### 2.2 The coefficient of thermal expansion of paving concrete and its significance

The CTE of paving concrete varies over a broad range depending on the constituent materials. As coarse aggregate accounts for 50% to 60% of the volume of concrete thus the mineralogy of coarse aggregate is the most significant factor affecting CTE of concrete. The average CTE values of paving concrete with regards to the coarse aggregate/gravel type are shown in Table 2, which clearly shows the effect of gravel on the CTE of hardened concrete [13]. These values are the national average of extensive CTE testing which was conducted for various types of paving mixes under long-term pavement performance (LTPP) research program. J. Tanesi et al. [14] determined the impact of CTE variation on concrete pavement performance by performing a parametric analysis. They concluded that transverse cracking increases with increase in CTE value. Hein and Sullivan [15] indicated that thermal movements of concrete pavement slab have significant effects on its performance including cracking and faulting. The CTE of overlay slab effects the UBCO performance as the thermal gradient exists in top 254 mm of the pavement and below that there is constant temperature thus the CTE of the underlying concrete layer has minimal effect and is generally neglected.

**Table 2:** Effects of coarse aggregate/gravel type on CTE of paving concrete [9].

Coarse aggregate/gravel type	Average CTE of concrete (micro-strain/°C)	Average CTE of concrete (micro-strain/°F)
Andesite	7.78	4.32
Basalt	7.79	4.33
Chert	10.82	6.01
Diabase	8.35	4.64
Dolomite	8.91	4.95
Gabbro	7.99	4.44
Gneiss	8.76	4.87
Granite	8.49	4.72
Limestone	7.81	4.34
Quartzite	9.34	5.19
Rhyolite	6.91	3.84
Sandstone	9.57	5.32
Schist	7.97	4.43
Siltstone	9.04	5.02

## 3 Methodology

Simulations were conducted in pavement ME version 2.3 for analysis of unbonded JPCP overlay of existing/deteriorated JPCP. The software requires various inputs including design life, traffic volume, overlay structure, concrete thermal and strength properties, properties of sub-layers, and condition of existing JPCP. To evaluate the impact of CTE variability, all the design inputs were considered constant for entire simulation work except the CTE of overlay concrete. The traffic and climatic details were considered according to the studies conducted by Hasan et al. [16, 17]. Primary design inputs are shown in Table 3.

**Table 3:** Design inputs for simulation.

Parameter	Value
Design life	30 years
JPCP overlay thickness	203 mm (8 in)
Dowel diameter	25 mm (1 in)
Joint spacing	4.57 m (15 ft)
Traffic (AADTT)	4000
Traffic (ESALS)	29 × 10^6^
Initial IRI	99 cm/km (63 in/mile)
IRI threshold	271 cm/km (172 in/mile)
Transverse cracking threshold (% Slabs)	15%
Joint faulting threshold	3 mm (0.12 in.)
Reliability	90%
Modulus of rupture of overlay concrete	4.75 MPa (690 psi)
Elastic modulus of overlay concrete	28.9 GPa (4.2 × 10^6^ psi)
Water to cement ratio in overlay concrete	0.42
HMA (Interlayer) thickness	50.8 mm (2 in)
HMA binder grade	PG 64-22
Existing JPCP thickness	203 mm (8 in)
Distressed elastic modulus of existing JPCP	16.8 GPa (2.44 × 10^6^ psi)
Base course	Non-stabilized (A-1-a)
Base course thickness	152 mm (6 in)
Base course resilient modulus	275 MPa (40000 psi)

According to Table 2, the CTE of concrete varies in the range of 6.8–10.8 micro-strain/°C (3.8–6 micro-strain/°F) and different CTE values were simulated within this range and the performance predictions of UBCO were analyzed. Furthermore, to contrast between different climatic conditions, the simulations were conducted for two climatic regions i.e. Albuquerque, NM with lower freezing index and a lesser number of freeze/thaw cycles considered as moderate climate region and Alamosa, CO with higher freezing index and a higher number of freeze/thaw cycles considered as cold region. The climatic details of both the regions are given in Table 4. The effects of varying CTE values on transverse cracking, joint faulting, and IRI were analyzed.

**Table 4:** Climatic details of simulated regions.

	Albuquerque, NM	Alamosa, CO
Mean annual air temperature (°C)	14.3	5.9
Mean annual precipitation (mm)	229.8	150.4
Freezing index (°C – days)	23.7	707.8
Average annual freeze/thaw cycles	75.85	187.82
Remarks	Moderate Region	Cold Region

## 4 Results and discussions

### 4.1 Impact of CTE variation on transverse cracking

**Fig. 1:**
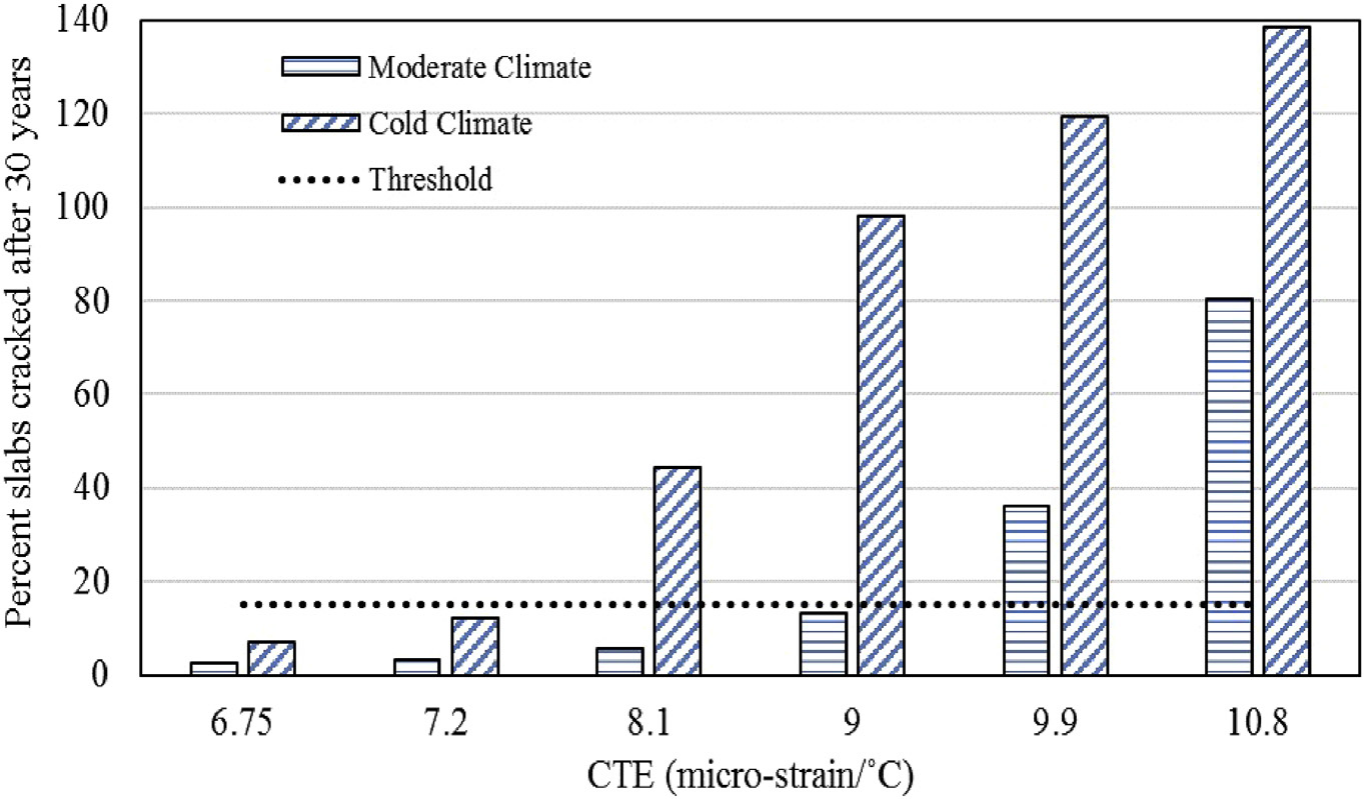
Effects of CTE on transverse cracking.

The analysis of CTE variation on the transverse cracking parameter of UBCO was conducted. The comparison of results of transverse cracking after 30 years of service life for both the climatic regions is presented in Fig. 1. This shows a significant increase in cracking distress with an increase in CTE of overlay concrete with all other design variables being constant. The ME design equations being used for quantifying the cracking distress has the main component of critical slab stress and increase in CTE leads to higher curling creating void spaces beneath the slab. This results in higher stresses due to traffic loading at the top or bottom center of the pavement slab depending on the type of curling (upward or downward curling) which transforms into higher transverse cracking.

For the moderate climatic region, the overlay fails to meet the design criteria of transverse cracking at the age of 17 years when CTE value reaches 9.9 micro-strain/°C (5.5 micro-strain/°F) and with further increase in CTE values the overlay fails even earlier. For the cold region, the overlay fails to meet the transverse cracking threshold at the life of 13 years with the CTE value of 8.1 micro-strain/°C (4.5 micro-strain/°F). The analysis shows that transverse cracking increases as the CTE of overlay concrete increases and the effect is more pronounced in the cold climate. The general trend of the decline in overlay performance confirms that higher CTE is associated with higher curling resulting in higher lift off from the pavement slab which results in higher stresses in the pavement slab due to the combined effect of traffic loading and curling. The significant difference in the overlay performance between the two climatic regions can be linked with many climatic factors including precipitation, humidity, number of freeze-thaw cycles and temperature variation pattern. With regards to CTE, the temperature variation pattern is the most critical factor. The average variation between day and night temperature for the moderate region is 6.6 °C (44 °F) and for the cold region, it is 15.5 °C (61 °F). As the day/night temperature variation results in the temperature gradient through the thickness of the pavement slab and higher temperature difference corresponds to a higher temperature gradient resulting in higher curling. Higher curling combined with traffic loading results in higher stresses and early deterioration of the pavement. The variation in performance can also be linked with the number of freeze/thaw cycles as loading/traffic volume and other design variables remained constant for these simulations. The cold region has a much higher number of freeze/thaw cycles in comparison with the moderate climatic region and the overlay pavement undergoes much more cracking in the cold region than in the moderate region.

To minimize the performance issues, either lower CTE concrete should be used or the pavement structure needs to be revised/modified for the higher CTE concrete so that overlay can perform within the prescribed performance parameters.

### 4.2 Effects of CTE variation on joint faulting

**Fig. 2:**
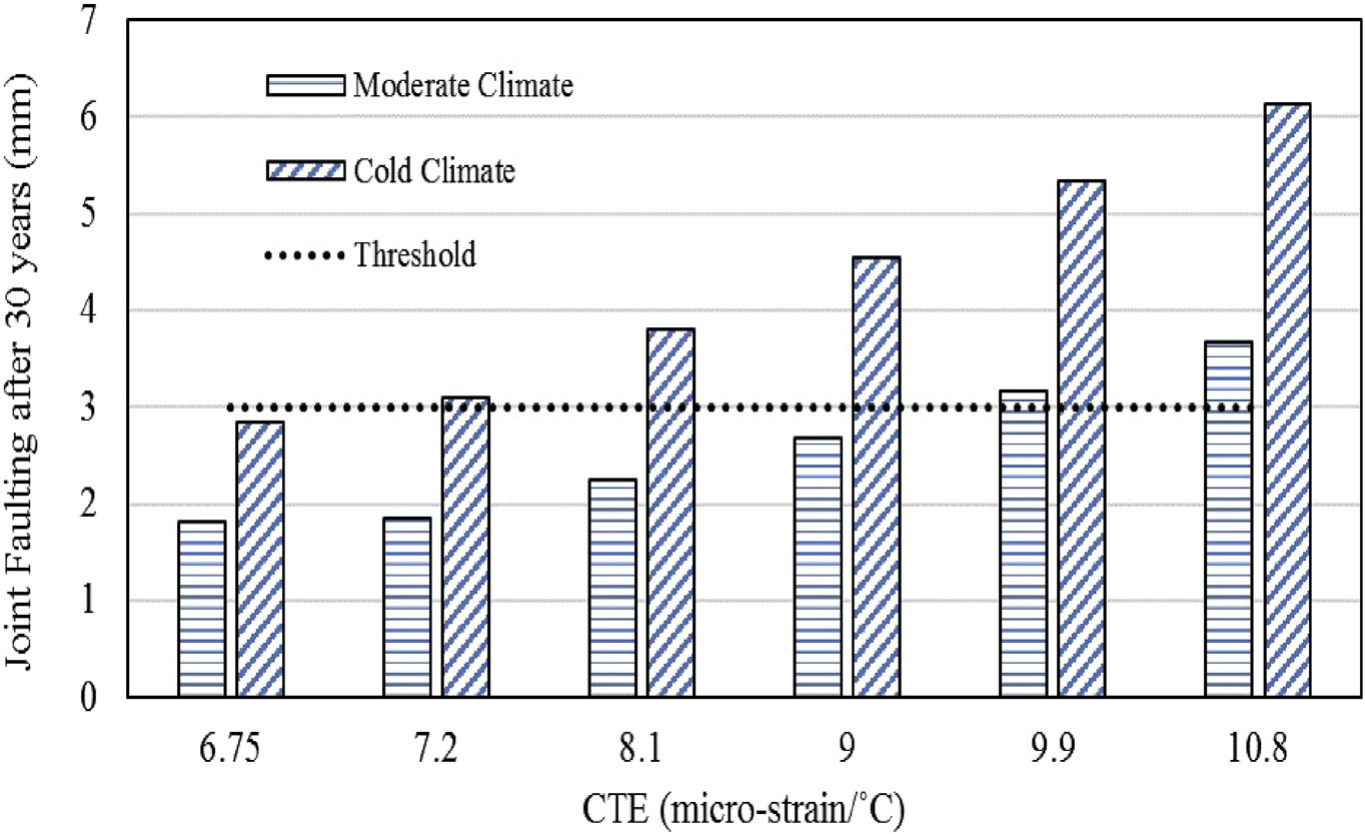
Effects of CTE on terminal joint faulting.

The impact of CTE variation on the performance of overlay with regards to joint faulting was analyzed and the results for both the climatic regions are presented in Fig. 2. There is a continually increasing trend in joint faulting distress with an increase in CTE of overlay concrete for both the climatic regions with all other design variables being constant. For the moderate climatic region, the overlay fails prematurely (at the age of 28 years) per the prescribed joint faulting threshold when CTE reaches 9.9 micro-strain/°C (5.5 micro-strain/°F) while in the cold region, the overlay life is restricted to 20 years with a CTE value of 8.1 micro-strain/°C (4.5 micro-strain/°F). The increase in joint faulting with an increase in CTE of overlay concrete can be linked with many different factors but the most important one is the upward deflection of the pavement slab edge due to upward curling because of the temperature gradient. With higher CTE, there will be higher curling, higher upward deflection of slab transverse edges and higher joint faulting.

With regards to the variation of performance between the two climatic regions, the primary factor is the difference in the day/night temperature variation. As noted previously, the average variation between day and night temperature for the moderate region is 10 °C (50 °F) and 15.5 °C (60 °F) for the cold region. As the day/night temperature variation results in a temperature gradient through the thickness of the pavement slab and higher temperature difference corresponds to a higher temperature gradient resulting in higher curling. Thus, for the same CTE, higher joint faulting is observed for the cold region as compared to the moderate region.

### 4.3 Effects of CTE variation on pavement roughness

**Fig. 3:**
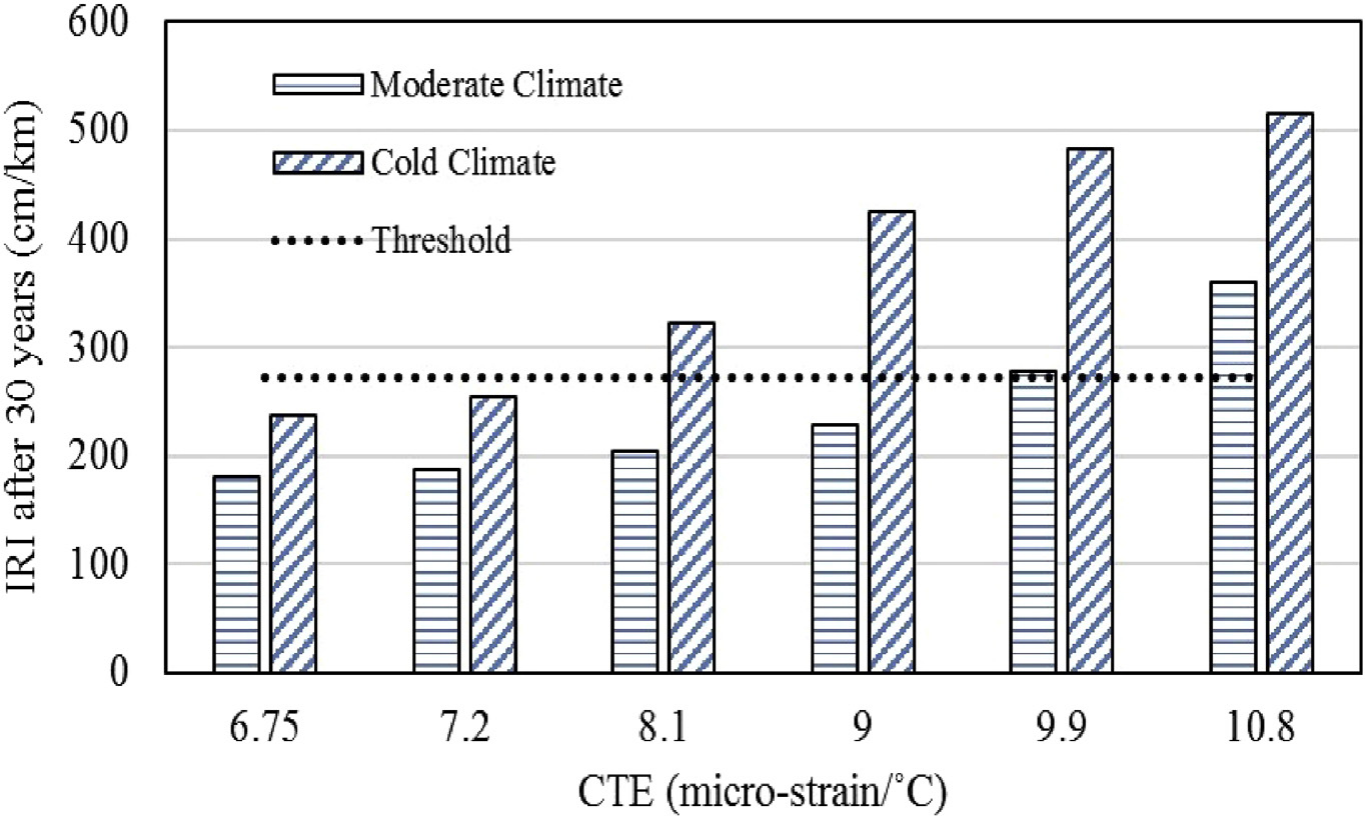
Effects of CTE on IRI.

The effects of CTE variation on the IRI were evaluated and the results for both the climatic regions are shown in Fig. 3. For the moderate climatic region, the overlay fails to meet the pavement roughness criteria at the age of 27 years when CTE reaches the value of 9.9 micro-strain/°C (5.5 micro-strain/°F). Whereas for the cold region the overlay fails in roughness at the age of 22 years with a CTE value of 8.1 micro-strain/°C (4.5 micro-strain/°F). With further increase in CTE of concrete, the overlay service life decreases further.

The decline in the performance of UBCO with regards to pavement roughness with an increase in CTE of overlay concrete is principally attributed to higher transverse cracking and higher joint faulting while other design variables being constant. As discussed earlier in this paper, cracking and faulting distresses increase with an increase in concrete CTE thus, IRI also increases with increase in CTE.

For different climatic regions, there is another crucial factor of freezing index in the performance prediction model of pavement ME design. The higher freezing index will result in higher IRI and the same trend has been observed in the simulation results as the overlay pavement in the cold region experienced higher pavement roughness due to higher freezing index as compared to the moderate region with lower freezing index. To mitigate the effects of higher CTE and to improve the overlay performance the overlay design needs to be modified to achieve the ultimate design life.

### 4.4 Sensitivity analysis of performance parameters

**Fig. 4:**
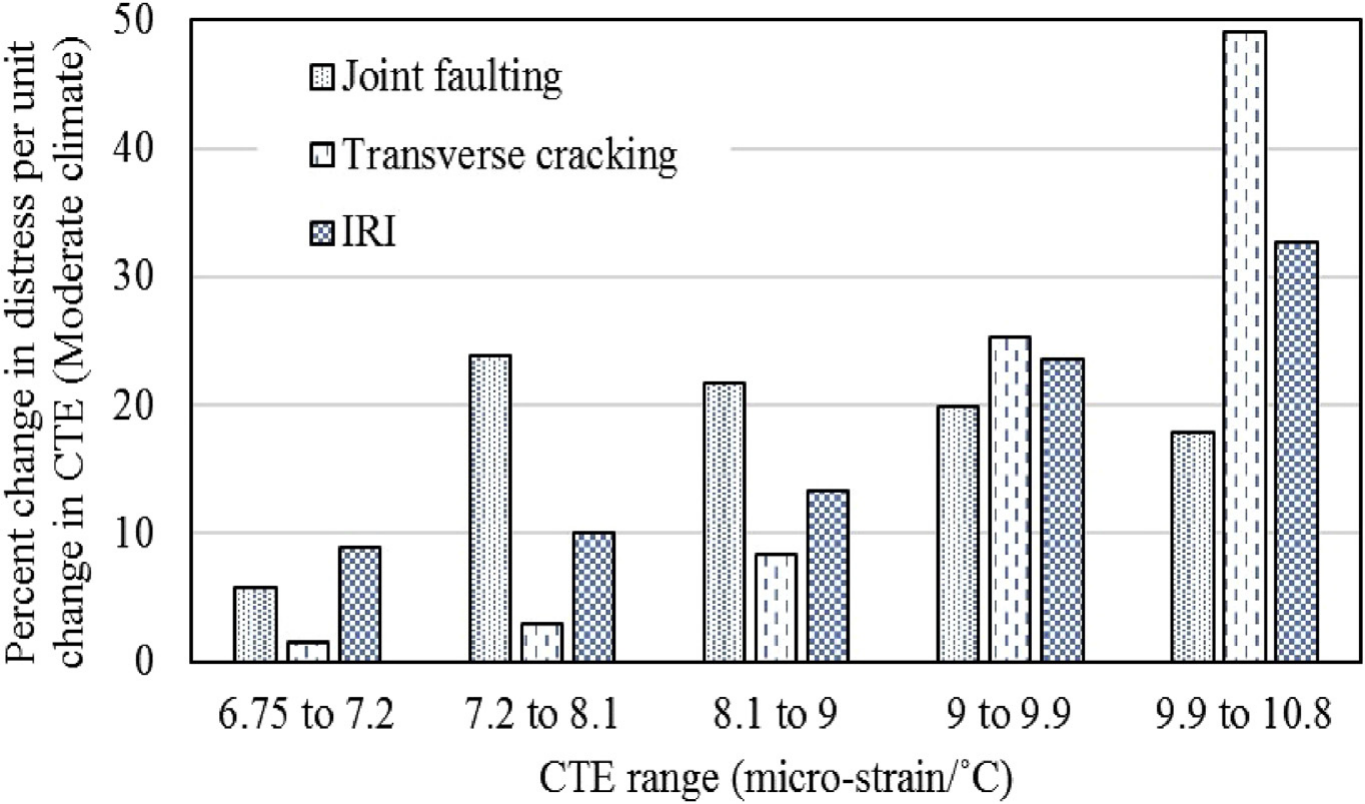
Sensitivity analysis for moderate climate.

**Fig. 5:**
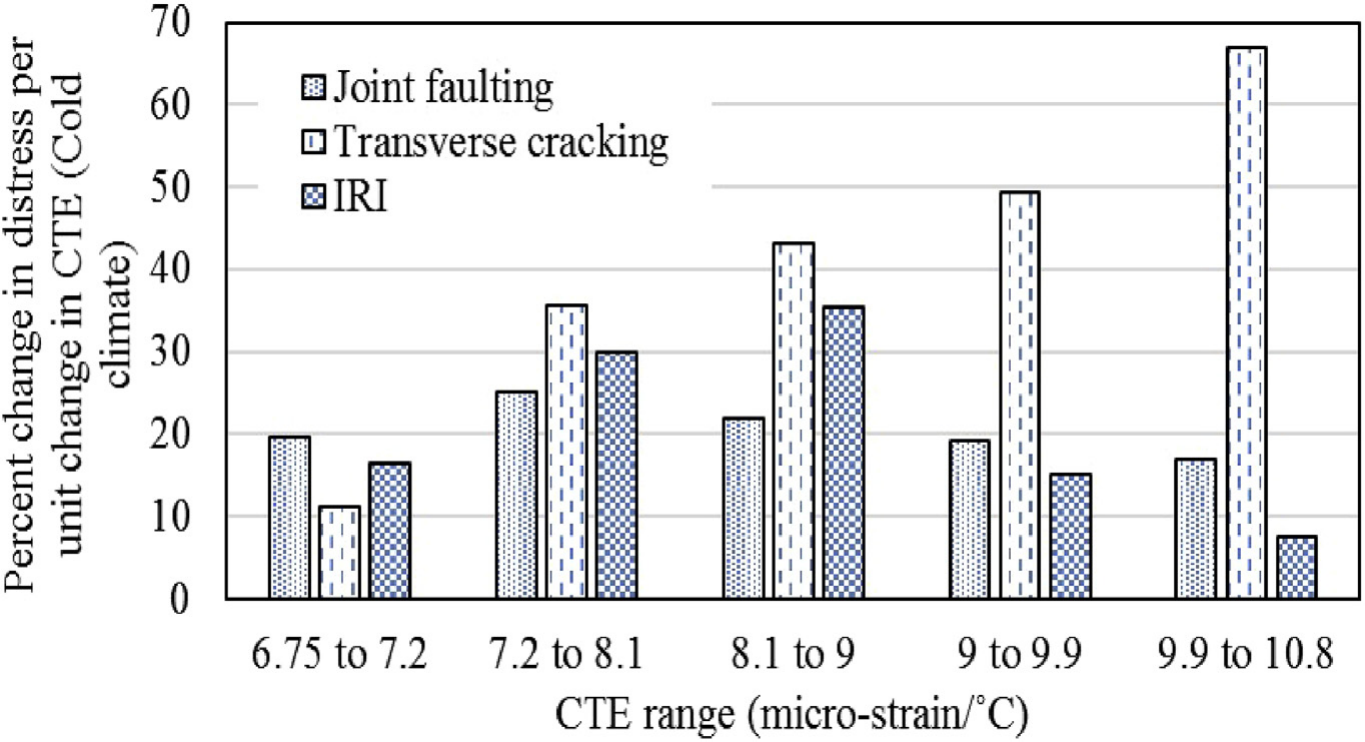
Sensitivity analysis for cold climate.

As variation in performance predictions was observed with varying CTE of overlay concrete thus, a sensitivity analysis was performed to quantify the impact of CTE increase on the three performance parameters and to determine the most significantly affected performance measure. The normalized percent change for cracking, faulting and IRI were obtained for a unit change in CTE for different ranges of CTE and the results are shown in Figs. 4 and 5. According to the results, the transverse cracking is the most affected parameter with a change in CTE of concrete with percent change of up to 49.3% for the moderate climate and up to 67.1% for the cold region. Joint faulting is the least affected performance parameter with percent change of up to 23% for the moderate climate and up to 25% for the cold region. Pavement roughness is a little more affected than the joint faulting. Another critical finding was observed regarding the transverse cracking predictions that when the transverse cracking reaches 100% (meaning that all the slabs are cracked) than the percent change in transverse cracking decreases because there is no significant meaning of more than 100% slabs cracked. Thus, it can be concluded that transverse cracking is the most affected performance parameter with CTE variation for both climatic regions. The factors responsible for this variation have already been discussed in the preceding section.

### 4.5 Minimizing the performance issues of higher CTE

According to the performance criteria set forth for this study, the UBCO fails if it does not comply with the prescribed performance threshold until the end of design life. As evident from the results shown earlier that many design simulations do not meet the performance standards, both in the moderate climatic region and cold region. Many different measures can be taken to improve the overlay performance and to mitigate the effects of higher CTE and to ensure that the overlay pavement can perform up to the designed life. These measures include increased overlay thickness, reduced joint spacing, increased dowel size, widened slabs, enhancing concrete strength properties, and tied shoulders. For this study, two of these measures were considered for further evaluation with regards to transverse cracking, which is the most affected performance parameter. These were the reduction in joint spacing and increase in overlay slab thickness.

#### 4.5.1 Effects of joint spacing on transverse cracking

**Fig. 6:**
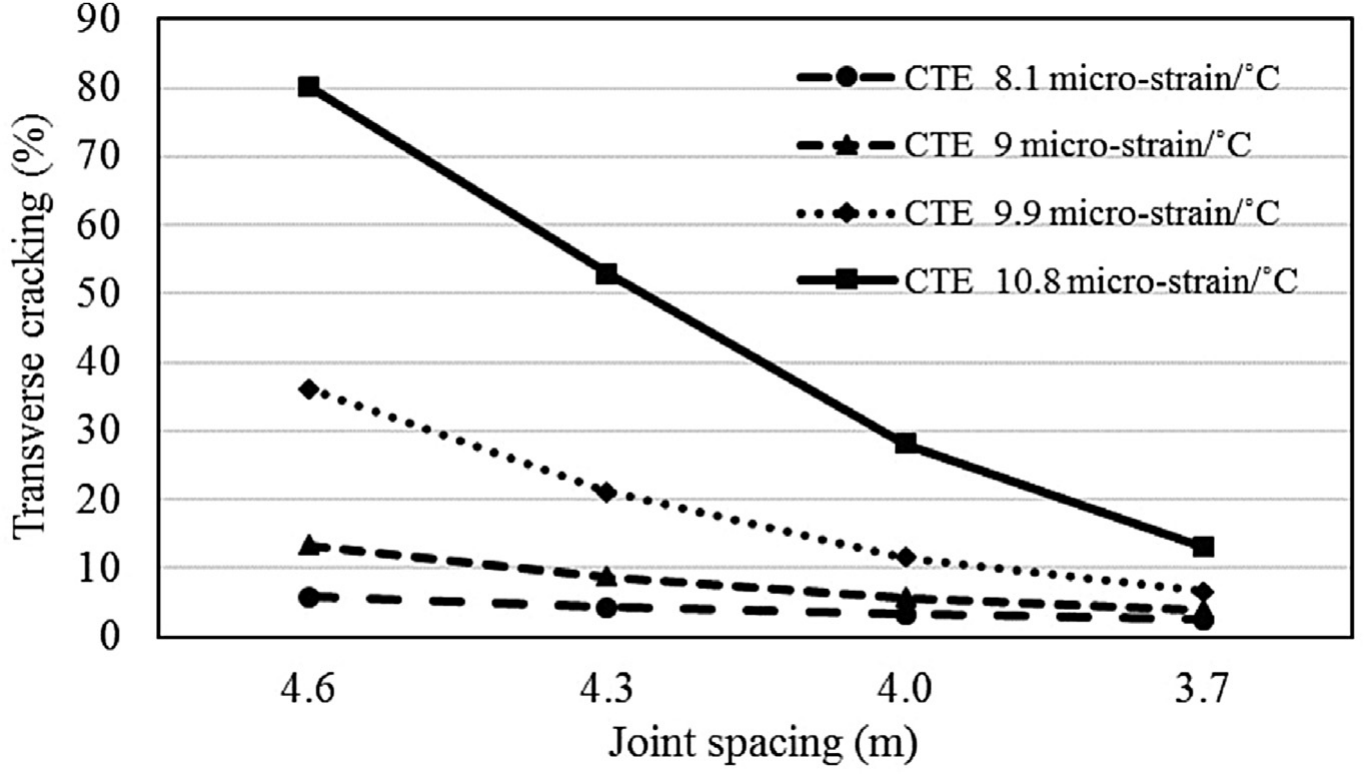
Impact of joint spacing on transverse cracking (moderate climate).

**Fig. 7:**
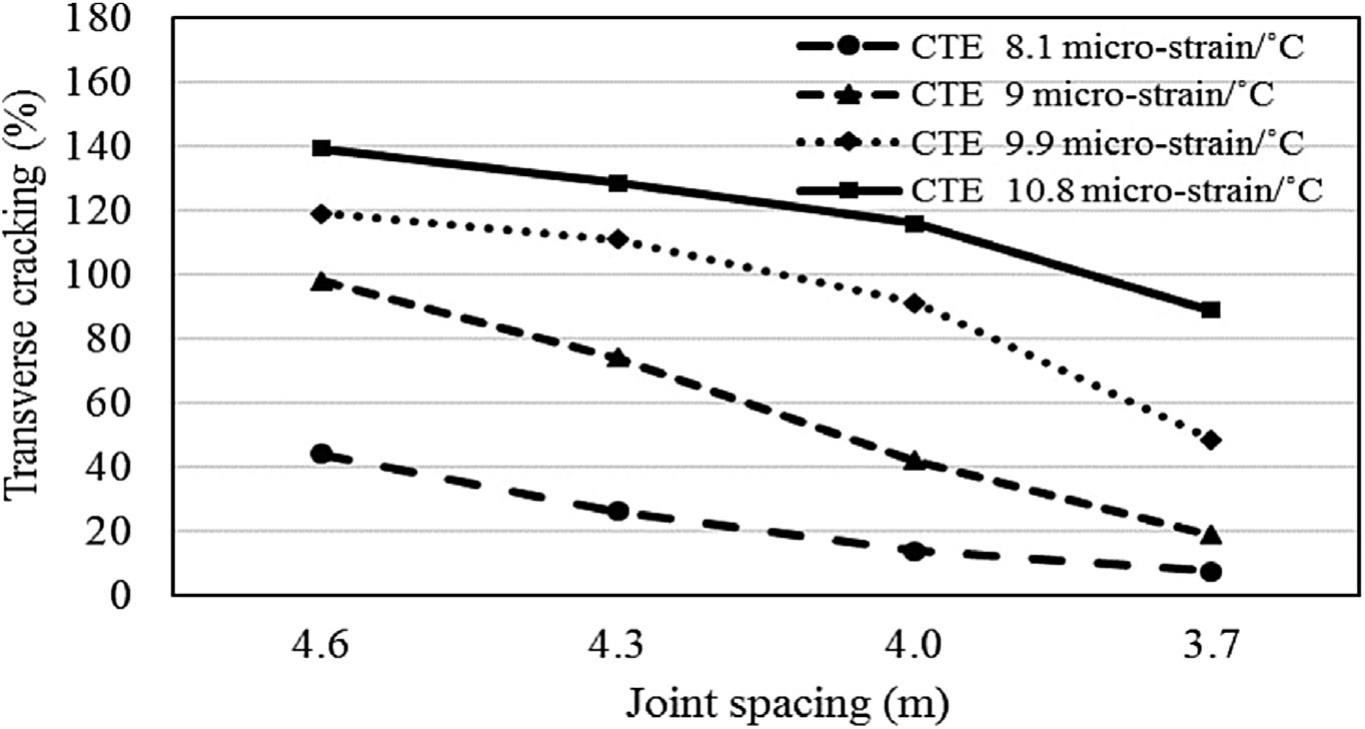
Impact of joint spacing on transverse cracking (cold climate).

Simulations were conducted for CTE range of 8.1–10.8 micro-strain/°C with a different transverse joint spacing of overlay slab to observe the impact of joint spacing on transverse cracking. The results are presented in Figs. 6 and 7 for the effects of joint spacing for both climatic regions. The results show a significant impact of joint spacing on the cracking performance and numerically varies for different CTE values. The highest improvement in cracking distress with 0.3 m reduction in joint spacing is 27% for CTE of 10.8 micro-strain/°C whereas the average improvement is around 8.5% for the moderate climate and 19.7% for the cold region. Thus, it is concluded that with a reduction in transverse joint spacing the adverse effects of higher CTE can be minimized.

#### 4.5.2 Effects of overlay thickness on transverse cracking

**Fig. 8:**
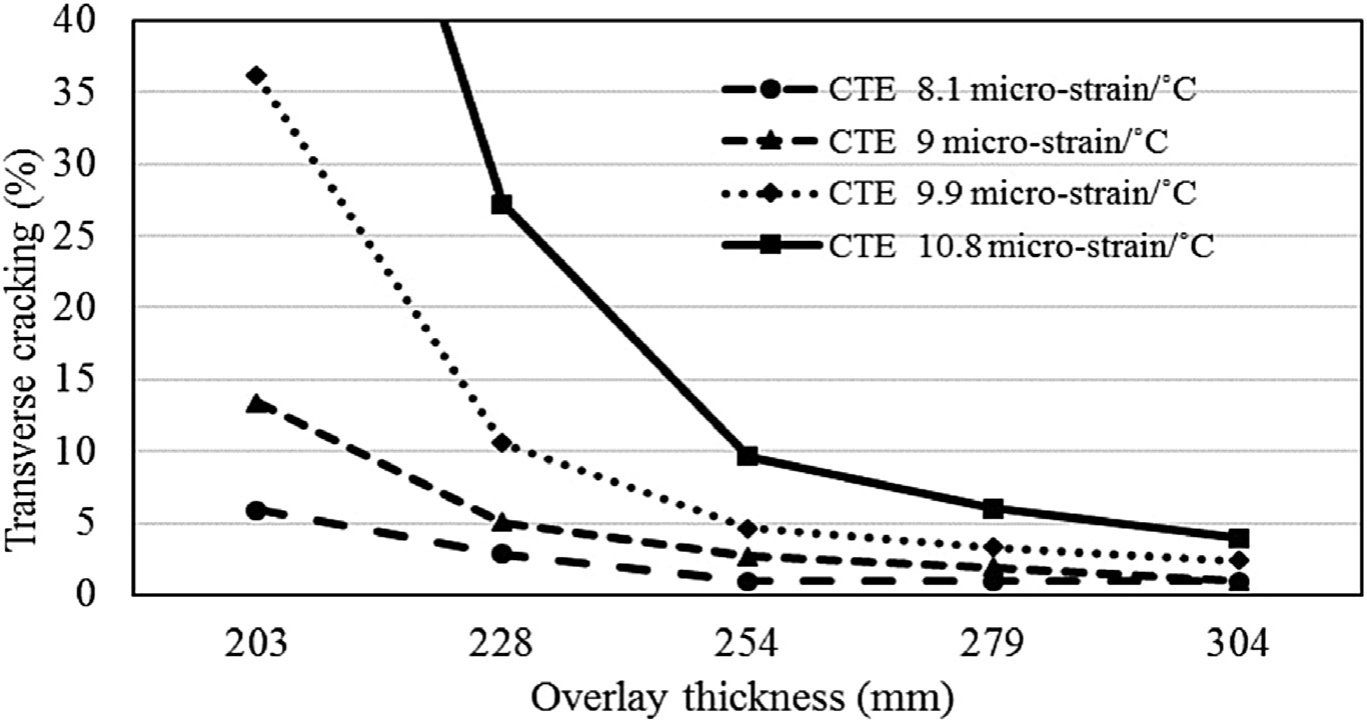
Impact of overlay thickness on transverse cracking (moderate climate).

**Fig. 9:**
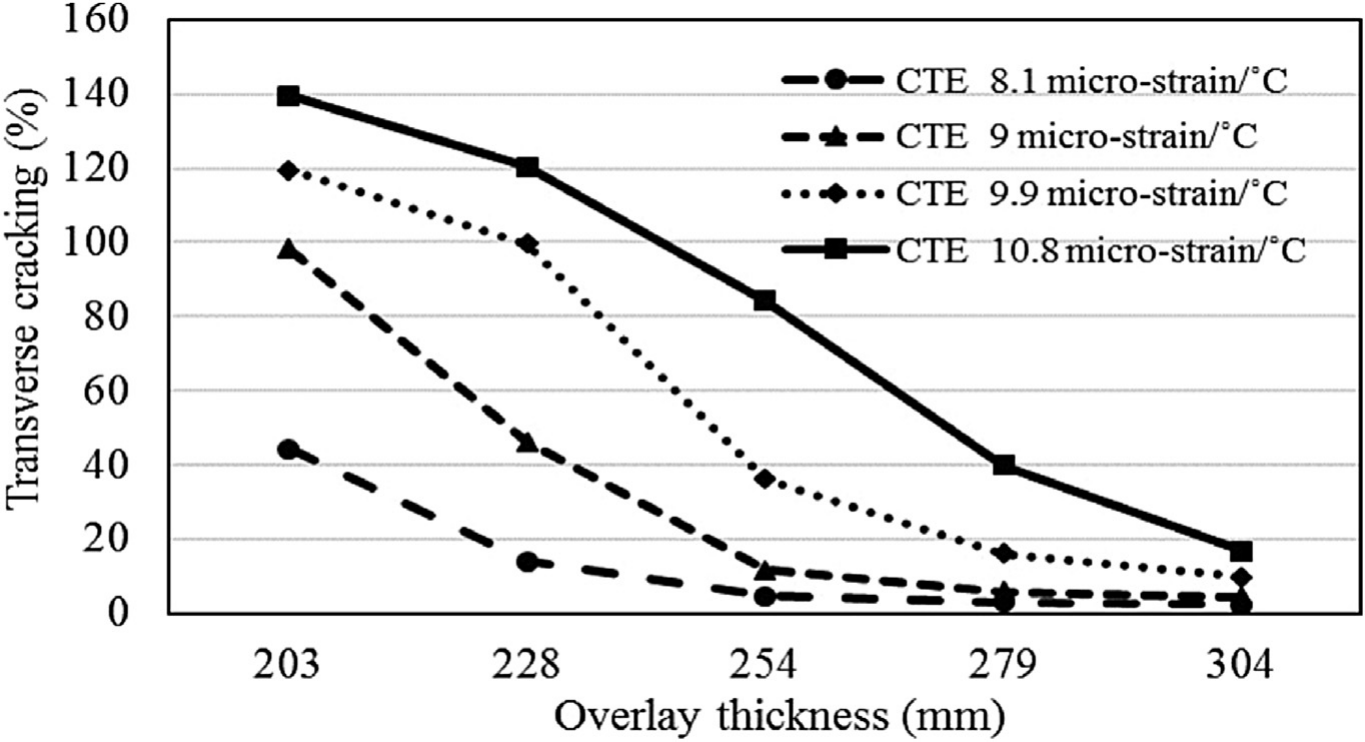
Impact of overlay thickness on transverse cracking (cold climate).

Revised simulations were conducted with various overlay slab thicknesses ranging from 203 to 304 mm to evaluate the effect of slab thickness on transverse cracking for different CTE values ranging from 8.1 to 10.8 micro-strain/°C. The comparison is presented in Figs. 8 and 9 for both the climatic regions. There is an average trend of improved performance with regards to transverse cracking with an increase in slab thickness for both the regions. The percent reduction in transverse cracking varies for different slab thicknesses and different CTE values. The average decrease in cracking with an increase in overlay thickness of 25 mm is 8% for the moderate region and 23% for the cold region. The maximum improvement in cracking is 53% for the moderate region with CTE of 10.8 micro-strain/°C and 63% for the cold region with CTE of 9.9 micro-strain/°C which proves that thicker overlay slabs can mitigate the effects of higher CTE.

### 4.6 Validation with field performance data

The simulation results were validated by comparison of the performance data of actual UBCO projects constructed in various parts of the United States under the Long-Term Pavement Performance (LTPP) program. Material properties and field performance data from five (5) unbonded jointed PCC overlays over JPCP pavements constructed under General Pavement Study (GPS)-9 were extracted from the LTPP database. This was an important step to validate the simulation results presented earlier. These projects were given case # A to E for data analysis and comparison. The location and construction details of these projects are given in Table 5.

**Table 5:** Details of UBCO sections used for validation.

Case#	State	County	Type	UBCO construction	Existing JPCP thickness (mm)	HMA layer thickness (mm)	UBCO thickness (mm)
A	California	Placer	Interstate	1988	193.0	25.4	223.5
B	Kansas	Shawnee	U.S-2	1987	223.5	50.8	147.3
C	Minnesota	Renville	U.S-2	1987	198.1	20.3	149.9
D	Pennsylvania	Clearfield	Interstate	1988	246.4	83.8	261.6
E	Texas	Ellis	Interstate	1989	251.5	35.6	261.6

The overlay concrete of these projects had varying material properties including compressive strength, elastic modulus, flexural strength, and CTE. The CTE values of overlay concrete ranged from 6.8 to 8.8 micro-strain/°C with different types of coarse aggregates. The traffic volume for these highways was also different for every project. These details are presented in Table 6.

**Table 6:** Overlay concrete properties and traffic volume.

Case#	Overlay concrete properties					Traffic (AADTT)
	MOR (MPa)	Compressive strength (MPa)	Elastic modulus (GPa)	CTE (μԐ/°C)	Coarse aggregate	
A	5.2	44.1	31.4	7.3	Lime stone	1129
B	6.1	60.1	27.6	7.4	Lime stone	399
C	5.6	49.7	36.5	8.2	Granite	140
D	3	25.8	31.3	8.8	Granite	2378
E	6.3	63.5	37.7	6.8	Lime stone	1665

Based on the material properties, design properties, traffic volume, and climate data, simulations were conducted in pavement ME design software to obtain the performance predictions for these projects. Different climate stations were used for each of these projects based on their location. The field performance data of actual projects was obtained from the LTPP database and comparison was conducted with the simulation results. The comparison is presented in Table 7. The analysis showed that the results of ME design predicted performance for case A, C, D, and E were almost similar to the field performance values with a variation ranging up to 2.5%. The cracking prediction of case B was way lower than the simulation result with a difference of 38% which can be attributed to other factors such as construction flaws. As four of these projects matched closely with the simulated results of transverse cracking so it can be concluded that the simulation framework and procedure stand validated. The comparison of joint faulting predictions and field performance was conducted, and the results showed that four projects had a variation in joint faulting values ranging from 1.1 to 1.4 mm while case B again showed a higher difference of 2.7 mm between the simulation result and the field performance. The pavement roughness values also had a slight variation ranging from 0.6 to 0.9 m/km. These results show that pavement ME design software can accurately predict the performance of UBCO pavement systems.

**Table 7:** Comparison of field performance with simulation results.

Case#	Field performance				ME design predictions		
	Age (years)	Transverse cracks (%)	Faulting (mm)	IRI (m/km)	Transverse cracks (%)	Faulting (mm)	IRI (m/km)
A	14	0	2.1	2.4	1	1.0	1.6
B	7	39	3.2	2.1	1	0.5	1.4
C	8	3	1.6	2.1	2	0.5	1.5
D	14	8	0.1	0.8	5.5	1.5	1.8
E	26	0	0.2	0.9	0	1.3	1.6

## 5 Conclusions

• CTE of overlay concrete is directly related to the performance of unbonded concrete overlay and with an increase in CTE the overlay performance, including transverse cracking, faulting, and roughness, is adversely affected. Transverse cracking is the most affected parameter with the change in CTE as compared to pavement roughness and joint faulting. The major contributing factor of this phenomenon is a higher amount of slab curling and increased curling stresses due to higher CTE which consequences in void space beneath the slab, making the pavement slab un-supported that results in increasing stresses due to traffic loading. These adverse effects are more severe in cold climatic regions as compared to moderate climate region due to higher temperature gradient resulting in higher curling stresses. The climatic pattern including the variation in day/night temperature and freeze-thaw cycles also affect the performance of UBCO pavement as observed with the difference between the pavement performance predictions of moderate and cold climatic regions with all other design inputs as constant.
• The geometric properties of overlay pavement including transverse joint spacing and slab thickness have a direct impact on the performance parameters. The average decrease in cracking distress is around 8.5% for the moderate climate and 19.7% for the cold region with 0.3 m reduction in joint spacing. The average decrease in cracking with an increase in overlay thickness of 25 mm is 8% for the moderate region and 23% for the cold region. Thus it is evident that the overlay performance can be improved by increased overlay slab thickness or reduced joint spacing and with these modifications, the adverse effects of higher CTE can be compensated.

## Declarations

### Author contribution statement

Gauhar Sabih: Conceived and designed the analysis; Analyzed and interpreted the data; Contributed analysis tools or data; Wrote the paper.

Tahmidur Rahman, Rafiqul A. Tarefder: Contributed analysis tools or data; Wrote the paper.

### Funding statement

This research did not receive any specific grant from funding agencies in the public, commercial, or not-for-profit sectors.

### Competing interest statement

The authors declare no conflict of interest.

### Additional information

No additional information is available for this paper.
